# Effects of Haloperidol on Cardiac Histamine H_2_ Receptors and β-Adrenoceptors in Isolated Mouse and Human Atrial Preparations

**DOI:** 10.3390/neurosci6030091

**Published:** 2025-09-17

**Authors:** Jonas M. A. Schlicht, Britt Hofmann, Uwe Kirchhefer, Joachim Neumann, Ulrich Gergs

**Affiliations:** 1 Institute for Pharmacology and Toxicology, Medical Faculty, Martin Luther University Halle-Wittenberg, 06097 Halle (Saale), Germany; j.schlicht@schlicht.net (J.M.A.S.); joachim.neumann@medizin.uni-halle.de (J.N.); 2 Department of Cardiac Surgery, Mid-German Heart Centre, University Hospital Halle, 06097 Halle (Saale), Germany; britt.hofmann@uk-halle.de; 3 Institute of Pharmacology and Toxicology, University Hospital Münster, University of Münster, 48149 Münster, Germany; kirchhef@uni-muenster.de

**Keywords:** haloperidol, histamine H_2_ receptor, heart, inotropy, chronotropy

## Abstract

The antipsychotic drug haloperidol is found on the WHO list of essential drugs. In vitro, haloperidol demonstrates binding affinity for various receptors, including histamine H_2_ receptors (H_2_Rs). Several cardiac effects of haloperidol are known, but it remains unclear whether H_2_Rs are involved. Here, the hypothesis was tested that haloperidol has the potential to act as either an agonist or an antagonist of human cardiac H_2_Rs. The contractile effects of haloperidol were studied in isolated left and right atrial preparations from transgenic mice overexpressing human H_2_Rs in the heart (H_2_-TG), and compared to human atrial preparations from adult patients. Haloperidol reduced the histamine-stimulated force of contraction in the human atrial preparations as well as the histamine-stimulated force of contraction and beating rate in the left and right atrial preparations from the H_2_-TG, respectively. Moreover, haloperidol reduced the isoprenaline-stimulated force of contraction in the human atrial preparations. In the wild-type mouse preparations, haloperidol only reduced the isoprenaline-stimulated beating rate in the right atria, but not the force in the left atria. Principally, haloperidol is capable of acting as an antagonist of both H_2_Rs and β-adrenoceptors in the human heart. However, the effects are only relevant at very high doses of haloperidol, which are never or seldom achieved in practice.

## 1. Introduction

**Figure 1:**
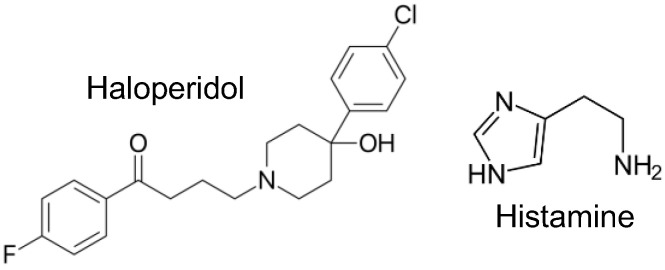
Structural formula of the compounds studied.

Haloperidol (Figure 1), a derivative of butyrophenone [1], was initially developed from compounds related to meperidine, a synthetic opioid, but which have no effect on opioid receptors (review [2]). The prefix “halo” indicates the presence of one or more halogens in the chemical structure of haloperidol. Haloperidol contains a fluorine atom on the phenyl ring and a chlorine atom on the butyrophenone ring (Figure 1). Haloperidol turned out not to be an analgesic drug but an antipsychotic drug [3]. In other words, haloperidol was not designed to act on monoamine receptors, and the biochemical basis of the psychiatric diseases treated by haloperidol is not yet fully understood. In this regard, parallels can be drawn with another antipsychotic agent, clozapine, which also exhibits an affinity for various neurotransmitter receptors and whose mechanism of action remains to be fully elucidated [4]. However, there is a consensus regarding the involvement of the dopaminergic pathway [5]. Therefore, it is conceivable that haloperidol acts as a neuroleptic agent because it is an antagonist of D_2_-dopamine receptors and 5-HT_2A_-serotonin receptors in the brains of patients [1]. In addition, haloperidol has been reported to be efficacious in pain management by either blocking dopamine receptors, modulating the NMDA pathway, or interacting with other receptors [6]. Furthermore, several randomized controlled trials have investigated the role of haloperidol as part of the management of delirium in intensive care unit patients [7]. Finally, haloperidol is found on the WHO list of essential drugs [8]. Although a recent meta-analysis showed that haloperidol has only minor adverse effects on the heart, cardiac arrhythmias associated with haloperidol are known, including QT prolongation and torsades de pointes [9,10]. Therefore, further studies on haloperidol and its effects on the human heart can be regarded as clinically relevant. The cardiac effects of histamine have been the focus of our research for a considerable time. Consequently, we were intrigued to observe that haloperidol also demonstrates a binding affinity to histamine H_2_ receptors (H_2_Rs) in appropriately transfected cells [11].

The cardiac (side) effects of haloperidol are well studied in animals. Haloperidol inhibits the current through rat cardiac L-type calcium channels (LTCCs; [12]). This could explain why a negative inotropic effect of haloperidol has been observed in animals (e.g., negative inotropic effect of 10 nM haloperidol in an isolated perfused rabbit heart), but not yet in human cardiac preparations [13]. Additionally, haloperidol can induce the release of Ca^2+^ from the endoplasmic reticulum, which has been suggested to lead to Ca^2+^-associated cell death, for example, in human astrocytes [14]. Haloperidol antagonized the positive inotropic effects of dopamine in isolated muscles strips from human ventricles [15]. The authors suggested that dopamine might have acted on D_1_ receptors or directly or indirectly via noradrenaline release on β-adrenoceptors, and that these effects might have been antagonized by haloperidol in human ventricles, but this has not been clearly shown [15].

Currently, four histamine receptors are known, labeled chronologically as H_1_-, H_2_-, H_3_-, and H_4_-histamine receptors, which are coded by different genes [16]. All four histamine receptor subtypes have been described at the functional level and at the RNA and/or protein level in the human heart [17,18]. Moreover, H_1_ and H_2_ receptors have been reported to be involved in pathophysiological processes, including cardiac arrhythmias and heart failure. In this context, H_1_R and H_2_R antagonists are being discussed as therapeutic modalities for a range of cardiac diseases [19].

However, in the hearts of mice, rats, dogs, and cats, histamine exerts its effects by releasing noradrenaline from cardiac stores. Subsequently, the release of noradrenaline leads to the stimulation of adrenergic receptors within the heart, resulting in an augmentation of its force of contraction (FOC) [18]. In the human heart (atrium as well as ventricle), histamine induces an increase in the FOC. These effects are antagonized by cimetidine and are therefore classified as H_2_R mediated [20,21,22,23,24]. To obtain a model for human cardiac H_2_Rs, a transgenic mouse with cardiomyocyte-specific overexpression of human H_2_Rs has been established and characterized (H_2_-TG; [25]). Using this model, it has been shown, for instance, that amitriptyline, an antidepressant that probably acts as an uptake inhibitor for monoamines in the human brain, is a pharmacological antagonist of H_2_Rs in H_2_-TG [26]. In addition, in human atrial preparations (HAPs), amitriptyline was demonstrated to be an antagonist of H_2_Rs [26]. However, to the best of our knowledge, contractile effects of haloperidol in HAPs via inhibition or stimulation of H_2_Rs or of β-adrenoceptors have not yet been reported.

Among the four known histamine receptors, the H_2_R is functionally expressed in both the human heart and our transgenic mouse model. Although haloperidol has been demonstrated to bind to this receptor, there is no data in the literature regarding its effects on H_2_ receptor- or β-adrenoceptor-mediated cardiac contraction. The objective of this study is to address this knowledge gap by investigating whether haloperidol acts on the H_2_ receptor as a partial agonist or antagonist and how this affects cardiac muscle contraction. A congress abstract including parts of this study has been published recently [27].

## 2. Materials and Methods

### 2.1. Transgenic Mice

The investigation was in line with the Guide for the Care and Use of Laboratory Animals published by the National Research Council [28]. The breeding, keeping, and handling of the mice complied with local legal requirements. The animals were kept according to approved protocols of the Animal Welfare Committee of Martin-Luther-University of Halle-Wittenberg, Germany. The H_2_-TG mouse line has been described before [25].

### 2.2. Contractile Studies in Mice

Contractile studies were performed as repeatedly described in more detail. In brief, wild-type (WT) and transgenic (H_2_-TG) mice of both genders, aged about 200 days, were euthanized by rapid cervical dislocation without anesthesia. The thorax was opened, and the heart was mobilized and cut from the ascending aorta to make sure the right atrium was not damaged. We prepared the left atria (LA) and right atria (RA) from the WT and H_2_-TG mice and transferred them into organ baths. The force was recorded under isometric conditions [25]. The LA were stimulated (MDE, Munich, Germany) with rectangular pulses of 20 V for 5 milliseconds of signal duration by platinum electrodes in the organ baths. The signals were fed into a bridge amplifier, digitized and quantified by use of dedicated software on a personal computer (PowerLab system including the LabChart 8.0 software: ADInstruments, Oxford, UK). The bathing (Tyrode’s) solution of the organ baths contained 119.8 mM NaCI, 5.4 mM KCI, 1.8 mM CaCl_2_, 1.05 mM MgCl_2_, 0.42 mM NaH_2_PO_4_, 22.6 mM NaHCO_3_, 0.05 mM Na_2_EDTA, 0.28 mM ascorbic acid, and 5.05 mM glucose. Ascorbic acid was used here to inhibit oxidation of isoprenaline. The solution was continuously gassed with 95% O_2_ and 5% CO_2_ and maintained at 37 °C and pH 7.4. Spontaneously beating the right atrial preparations from the mice (WT and H_2_-TG) was used to study any chronotropic effects.

Once equilibrium was reached, the following test series were carried out with the WT and H_2_-TG preparations (for the cumulative administration of substances, it was necessary to wait until the contraction force had reached a plateau under the influence of the previously administered substance):

(a) Propranolol (0.4 µM), followed by histamine (0.1 µM), followed by a haloperidol concentration–response curve (1–10 µM).

The following test series were carried out with the WT preparations:

(a) Incubation with 30 nM or 1 µM isoprenaline (time control);
(b) Preincubation with 30 nM or 1 µM isoprenaline, followed by a haloperidol concentration–response curve (1–10 µM).

### 2.3. Contractile Studies on Human Preparations

The contractile studies on the human preparations used the same equipment and Tyrode’s solution, as in the preceding paragraph and as described before [26,29]. The samples originated from 18 male patients and 7 female patients, 55–87 years old. The relevant comorbidities included congestive heart failure, coronary artery disease, arterial hypertension, atrial fibrillation, chronic kidney disease, obesity, and hyperlipidemia. The samples were transferred into Tyrode’s solution immediately after collection and transported to the laboratory for preparation within 30 min. After equilibrium was reached, histamine was added cumulatively (100 nM to 100 µM) to establish the concentration–response curves. Subsequently, 1, 3, and 10 µM haloperidol were added followed by 10 µM cimetidine. In other experiments, the concentration–response curves for histamine were generated with the prior addition of 1, 3, or 10 µM haloperidol. In the final experiments, 10 µM haloperidol was added after the 1 µM isoprenaline had reached a plateau. In some experiments, 50 mM potassium chloride was initially applied to depolarize the cardiac myocytes, resulting in altered excitability and allowing for evaluation of the role of Ca^2+^ channels in cardiac contractility. Subsequently, isoprenaline was added, followed by haloperidol. This was performed to assess the possible involvement of LTCC in the anti-adrenergic effect of haloperidol. This protocol has been used before [30]. Written informed consent was obtained from all the patients.

In brief, the following test series were carried out with the human preparations (for the cumulative administration of substances, it was necessary to wait until the contraction force had reached a plateau under the influence of the previously administered substance):

(a) The histamine concentration–response curve (10^−8^–10^−4^ M).
(b) Preincubation with 1 or 3 or 10 µM haloperidol, followed by the histamine concentration–response curve (10^−7^–10^−4^ M).
(c) The histamine concentration–response curve (10^−8^–10^−4^ M), followed by the haloperidol concentration–response curve (1–10 µM), followed by 10 µM cimetidine.
(d) Incubation with 1 µM isoprenaline (time control).
(e) Preincubation with 1 µM isoprenaline, followed by 10 µM haloperidol.
(f) Preincubation with 50 mM KCl, followed by 1 µM isoprenaline, followed by 10 µM haloperidol.

### 2.4. Data Analysis

The data shown in the figures are the means ± SEM. The statistical significance of the results was determined using an analysis of variance (ANOVA) followed by a post hoc test with a Bonferroni correction for multiple comparisons, or Student’s *t*-test for the comparison of two groups, using Prism 9.0 software (GraphPad Software, San Diego, CA, USA). A *p*-value < 0.05 was regarded as significant.

### 2.5. Drugs and Materials

Haloperidol was supplied by Research Biochemical Inc., Natick, MA, USA. Isoprenaline, histamine, and cimetidine were obtained from Sigma-Aldrich, Taufkirchen, Germany. Stock solutions of haloperidol and cimetidine were prepared in dimethyl sulfoxide (DMSO). Stock solutions of histamine and isoprenaline were prepared in water with ascorbic acid to protect against oxidation. All other chemicals were of analytical grade. Demineralized water was used throughout experiments for preparations of buffer. Stock solutions of drugs were freshly prepared daily.

## 3. Results

### 3.1. Interaction of Haloperidol and Histamine in Human Atrial Preparations

**Figure 2:**
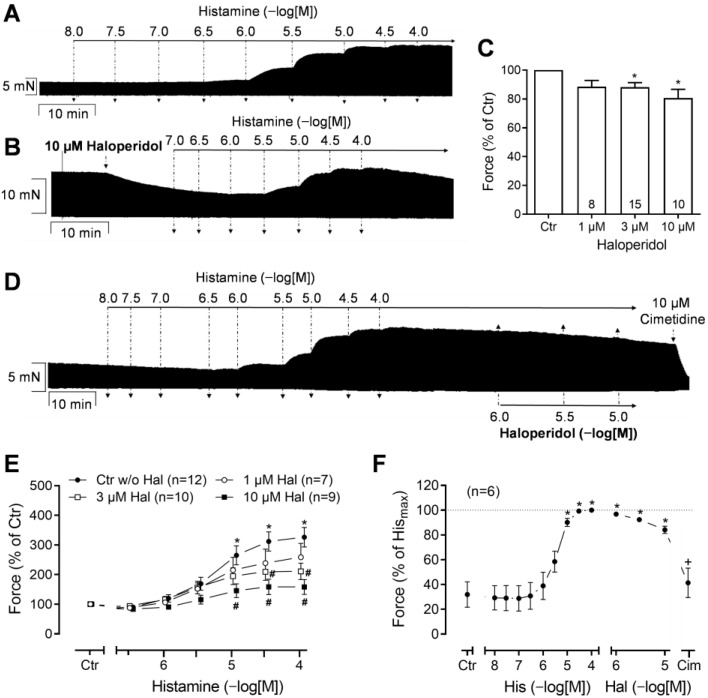
Force of contraction in human atrial preparations. (**A**,**B**,**D**) Exemplary original recordings: Positive inotropic effect of cumulatively applied histamine in human atrial preparations in absence (**A**) or in presence (**B**) of 10 µM haloperidol. (**D**) Haloperidol was cumulatively applied after concentration–response curve for histamine. (**C**,**E**,**F**) Summarized data: Numbers in brackets or bars indicate number of experiments. (**C**) Negative inotropic effect of haloperidol alone on force of contraction without any pre-stimulation. * *p* < 0.05 vs. Ctr (=pre-drug value). (**E**) Histamine-stimulated force of contraction normalized to pre-histamine values in absence (alone) or in presence of 1, 3, or 10 µM haloperidol (Hal). Force (100%) = 6.05 ± 1.14 mN. Negative decadic logarithm of 50% effective concentration (pEC_50_) for histamine: histamine alone (5.37); histamine in presence of 1 µM (5.33), 3 µM (5.71), and 10 µM (5.41) haloperidol. ^#^ *p* < 0.05 vs. histamine alone (closed circle). (**F**) Histamine-stimulated force of contraction normalized to maximum histamine (100 µM) values followed by negative inotropic effects of haloperidol and, finally, 10 µM cimetidine (Cim). Force (100%) = 10.52 ± 1.93 mN. * *p* < 0.05 vs. 100 µM histamine, ^+^ *p* < 0.05 vs. 10 µM haloperidol.

In the HAPs, the effect of histamine alone was investigated first. Histamine, cumulatively applied, exerted a positive inotropic effect (PIE). Each concentration was incubated until a plateau was reached (Figure 2A). This necessitated high concentrations of up to 100 µM histamine, which is in agreement with prior publications [18,20,21,23,24,31]. In contrast, haloperidol alone reduced the FOC in the HAPs in a concentration-dependent fashion. This can be seen from an original recording (Figure 2B) and from the quantified data in Figure 2C, indicating that the negative inotropic effect of haloperidol starts at 1 µM. As a time control, the time-dependent rundown of the developed force was calculated within a time frame of 20 min. In addition, since the haloperidol stock solution was prepared in DMSO, control experiments were performed with appropriately diluted DMSO, which demonstrated that the solvent had no effect on the force of contraction that developed (Supplementary Figure S1). After the negative inotropic effect of 1 or 3 or 10 µM haloperidol had reached a new plateau, histamine was added cumulatively again. A typical original recording of 10 µM haloperidol followed by its histamine concentration–response curve is shown in Figure 2B. The hypothesis was that each single concentration of haloperidol, applied before application of histamine, would concentration-dependently attenuate the PIE of cumulatively added histamine and shift this curve in a competitive or a non-competitive manner. Several such experiments are summarized in Figure 2E as percentage of the pre-drug value, i.e., the developed force immediately prior to application of the first histamine concentration. It should be noted that haloperidol exerted a downward shift on the concentration–response curve of histamine, suggesting a non-competitive antagonism. For comparison, the concentration-dependent PIE of histamine alone is also shown (“Ctr w/o Hal” in Figure 2E). This assumption was confirmed by the absence of a shift in the pEC_50_ values of histamine in the presence of increasing concentrations of haloperidol. The pEC_50_ values for histamine were as follows: 5.37 for histamine alone; 5.33, 5.71, and 5.41 for histamine in presence of 1 µM, 3 µM, and 10 µM haloperidol, respectively. Moreover, histamine alone is well known [29] to increase the maximum rate of tension development and the maximum rate of relaxation in HAPs, and this was also observed in the present set of experiments (Supplementary Figure S2). These effects were also attenuated by haloperidol in a non-competitive fashion (Supplementary Figure S2). There is a tendency of histamine to shorten the time to peak tension and time of relaxation, and this might have been reduced by the haloperidol, but it did not reach significance in this set of experiments (Supplementary Figure S2).

Thereafter, the mode of the addition of haloperidol was changed. First, histamine was cumulatively applied and increased the FOC in the HAPs. Subsequently, haloperidol decreased the histamine-induced FOC in a concentration-dependent manner (Figure 2D, original recording; Figure 2F, summarized data). This finding suggests that haloperidol functions as an antagonist rather than an agonist under these conditions, indicating that haloperidol is not a partial agonist of H_2_Rs in the HAPs. Likewise, histamine increased the rate of tension development and the rate of relaxation (Supplementary Figure S3), and these increases were also antagonized by the subsequently added haloperidol in a concentration-dependent manner (Supplementary Figure S3). Again, there was a tendency of histamine to reduce the time to peak tension and the time of relaxation, which did not reach significance (Supplementary Figure S3). Moreover, to confirm that the remaining effect of histamine was H_2_R-mediated, cimetidine, an H_2_R antagonist, was applied that could completely antagonize the PIE of histamine (Figure 2D,F). From these experiments, it could be concluded that haloperidol is a less effective antagonist than cimetidine of human H_2_Rs in HAPs, at least under these conditions.

### 3.2. Interaction of Haloperidol and Histamine in Mouse Atrial Preparations

**Figure 3:**
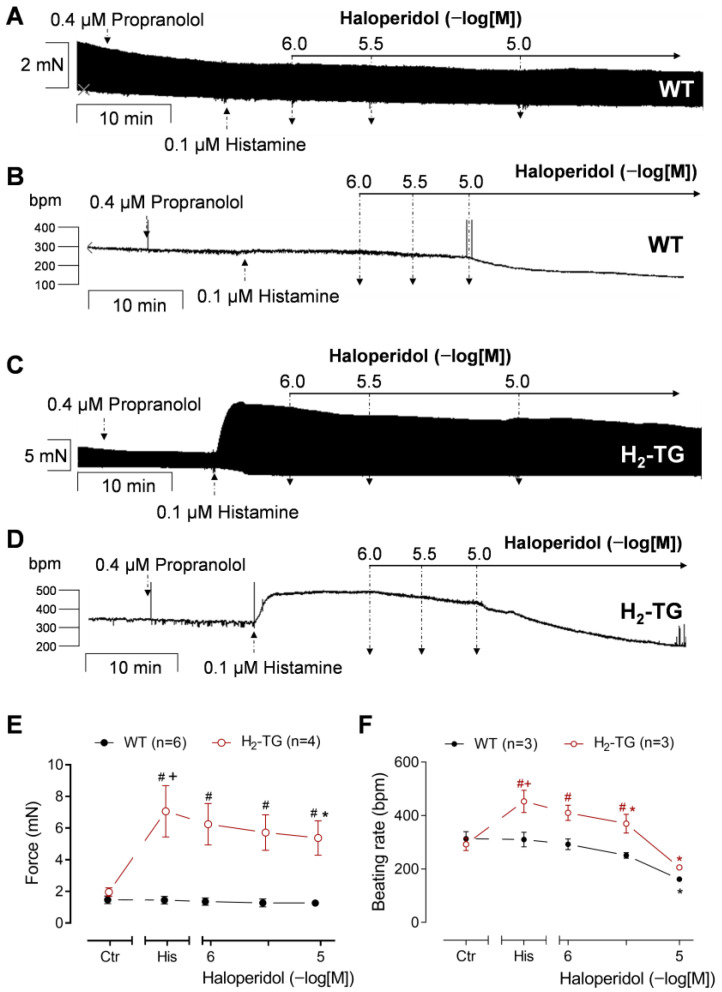
Effects of haloperidol in left and right atrial preparations of H_2_-TG in presence of histamine. Negative inotropic and chronotropic effects of 1, 3, and 10 µM haloperidol on H_2_-TG and wild-type (WT) mouse left and right atrial preparations, respectively, in presence of 0.1 µM histamine (His). Force of contraction or beating rate before addition of histamine, but in presence of propranolol, was designated as control value (Ctr). Numbers in brackets indicate number of experiments. Abscissae indicate concentrations of haloperidol in negative logarithmic molar concentrations. (**A**–**D**) Original recordings of inotropic (**A**,**C**) and chronotropic (**B**,**D**) effects of 1, 3, and 10 µM haloperidol in H_2_-TG mice (**C**,**D**) and WT mice (**A**,**B**) after histamine stimulation and preincubation with propranolol. (**E**,**F**) Summarized data: (**E**) Force of contraction in milli-Newton (mN) of electrically stimulated (1 Hz) left atrial preparations. (**F**) Beating rate in beats per minute (bpm) of spontaneously beating right atrial preparations. ^+^ *p* < 0.05 vs. Ctr; * *p* < 0.05 vs. His 0.1 µM; ^#^ *p* < 0.05 vs. WT.

The next step was to investigate how much these effects of haloperidol were due to its antagonism of H_2_Rs or whether antagonism of haloperidol at β-adrenergic receptors might also be a contributing factor. To this end, the experiments were modified in the following way: Now, atrial preparations from the H_2_-TG and WT mice were used instead of HAPs. Then, 0.4 µM propranolol was applied first to block the β-adrenoceptors in order to rule out any additional effect of noradrenaline that might be released by the subsequently applied histamine (reviewed in [17]). After an incubation time of 10 min, a plateau was reached in the force of contraction or beating rate. Subsequently, a single concentration of histamine was added to the electrically driven LA and spontaneously beating the RA from the WT (Figure 3A,B) or from the H_2_-TG (Figure 3C,D). In line with previous work, only in the H_2_-TG but not in the WT, 100 nM histamine increased the FOC or beating rate (Figure 3A,B vs. Figure 3C,D) [25]. Thereafter, haloperidol was applied in the presence of histamine and reduced the histamine-stimulated FOC in the H_2_-TG, but not in the WT, in a concentration-dependent manner (Figure 3C,E). In the RA, however, the administration of additional haloperidol resulted in a reduction in the beating rate in both the WT (Figure 3B,F) and H_2_-TG (Figure 3D,F), although this effect was less pronounced in the WT (Figure 3F). These data suggest an anti-H_2_ histaminergic effect of haloperidol in the sinus node, at least in the H_2_-TG. However, the negative chronotropic effect of haloperidol in the RA from the WT may have been complemented by an anti-β-adrenergic effect of haloperidol in the mouse sinus node (vide infra). The present findings indicate that haloperidol functions as an antagonist rather than an agonist of human H_2_Rs, at least in the mouse heart. In addition, histamine increased the maximum rate of contraction and relaxation and reduced the time to peak tension and the time of relaxation in the H_2_-TG (as described before in [25]) but not in the WT (Supplementary Figure S4). The effects on the time parameters remained unaffected by haloperidol (Supplementary Figure S4), likely due to the reduced efficacy of haloperidol in comparison to cimetidine in reducing the FOC.

### 3.3. Interaction of Haloperidol and Isoprenaline in Human Atrial Preparations

**Figure 4:**
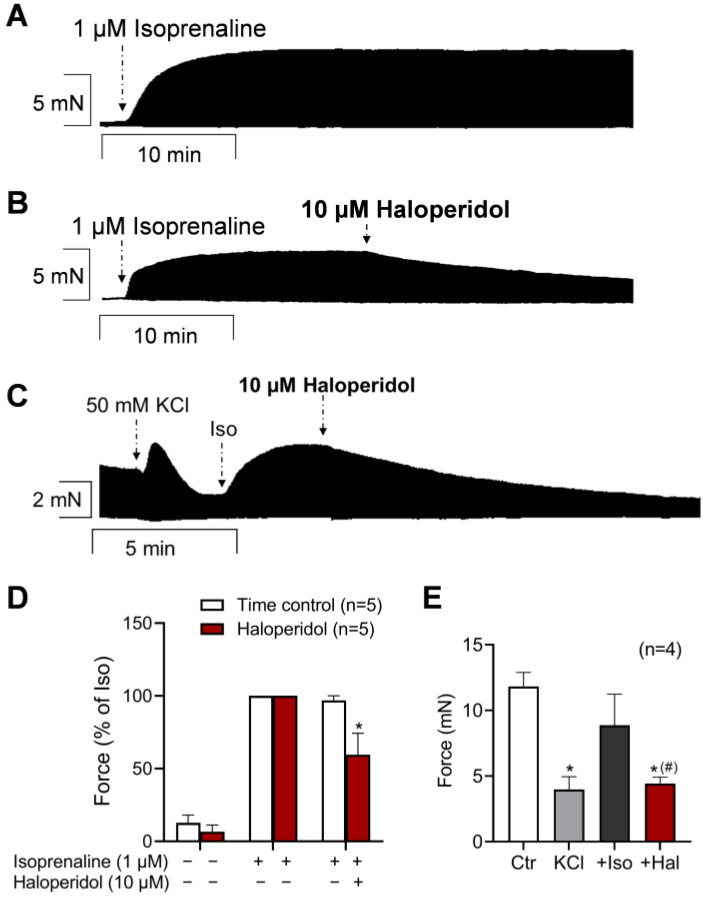
Effects of haloperidol on HAPs in presence of isoprenaline and in combination with potassium chloride and isoprenaline. Effect of 10 µM haloperidol (Hal) in presence of 1 µM isoprenaline (Iso) alone or after partial depolarization with 50 mM potassium chloride (KCl), followed by 1 µM isoprenaline in HAPs. Control value (Ctr) = Tyrode’s solution. (**A**,**B**) Original recording of negative inotropic effect of haloperidol in presence of 1 µM isoprenaline (**B**) in comparison to time control without addition of haloperidol (**A**). (**D**) Summarized data of force of contraction: Force (100%) = 8.00 ± 3.00 mN. Force (100%) time control = 7.72 ± 1.66 mN. The developed force before addition of haloperidol but in presence of isoprenaline was set to 100%. (**C**) Original recording: Prior to haloperidol application, HAP was incubated with 50 mM KCl followed by 1 µM isoprenaline (Iso). (**E**) Summary of negative inotropic effects of 10 µM haloperidol (Hal) in presence of 50 mM KCl and 1 µM isoprenaline. Force of contraction (100%) = 3.99 ± 0.97 mN. * *p* < 0.05 vs. 1 µM isoprenaline (ANOVA). ^(#)^ *p* < 0.05 vs. 1 µM isoprenaline (*t*-test). Numbers in brackets indicate number of experiments.

Histamine acts via cAMP in the heart, like isoprenaline. Hence, the effects of haloperidol on β-adrenoceptor stimulation of the cardiac preparations were studied for comparison. The administration of isoprenaline raised the FOC in the HAPs (Figure 4). Similarly, isoprenaline increased both the maximum rate of tension development and the maximum rate of relaxation (Supplementary Figure S5). After the force of contraction had reached a plateau again under the influence of isoprenaline, the subsequent administration of haloperidol reduced the FOC, as demonstrated in the original recording in Figure 4B (compared to the time control in Figure 4A) and as summarized in Figure 4D. Likewise, haloperidol reduced the isoprenaline-stimulated maximum rate of tension development and relaxation (Supplementary Figure S5). Hence, haloperidol also exerted anti-β-adrenergic effects in the HAPs.

### 3.4. Interaction of Haloperidol and Isoprenaline in Mouse Atrial Preparations

**Figure A1:**
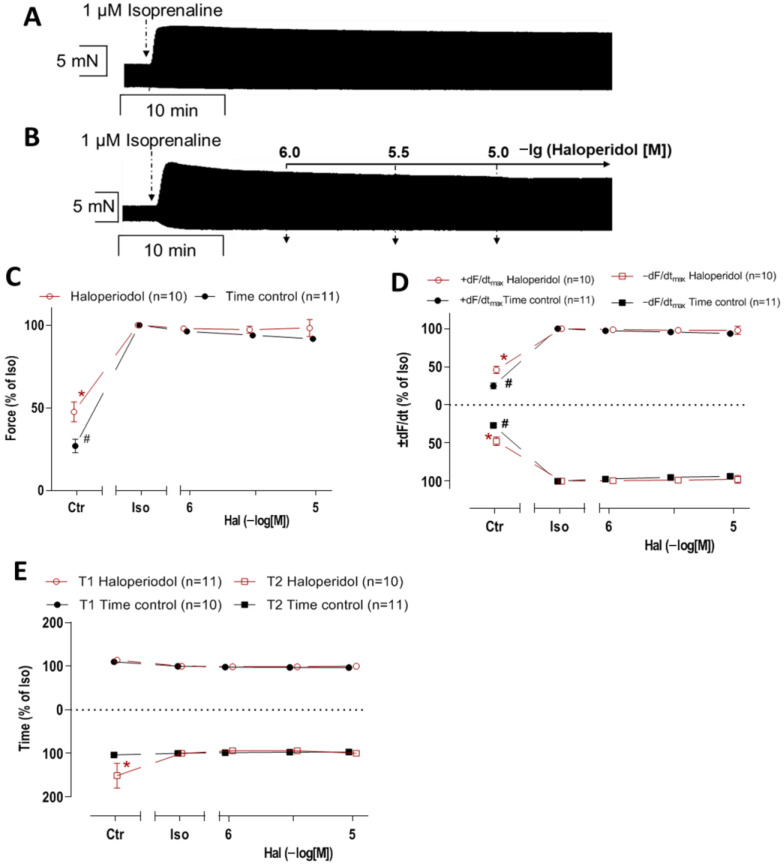
Effects of haloperidol in left atrial preparations of WT in presence of 1 µM isoprenaline. (**A**,**B**) Original recording of inotropic effect of 1, 3, and 10 µM haloperidol (Hal) on WT mouse left atria in presence of 1 µM isoprenaline (Iso) (**B**) in comparison to time control (**A**). (**C**) Force of contraction: force (100%) = 7.14 ± 1.06 mN; force time control (100%) = 8.86 ± 0.80 mN. (**D**) Maximum rate of contraction (+dF/dt_max_) and relaxation (−dF/dt_max_): +dF/dt_max_ (100%) = 404.90 ± 79.56 mN/s; +dF/dt_max_ time control (100%) = 507.40 ± 64.64 mN/s; −dF/dt_max_ (100%) = −255.24 ± 40.07 mN/s; −dF/dt_max_ time control (100%) = −304.30 ± 29.93 mN/s. (**E**) Time to peak tension (T1) and time to relaxation (T2): T1 (100%) = 17.47 ± 2.16 ms; T1 time control (100%) = 17.17 ± 2.05 ms; T2 (100%) = 31.34 ± 4.89 ms; T2 (100%) = 29.39 ± 2.19 ms. * *p* < 0.05 vs. Iso. ^#^ *p* < 0.05 vs. Iso (time control). Control value (Ctr) = pre-drug value. Reading before addition of haloperidol but in presence of isoprenaline was set to 100%. Numbers in brackets indicate number of experiments. Abscissae indicate concentrations of haloperidol in negative logarithmic molar concentrations.

**Figure A2:**
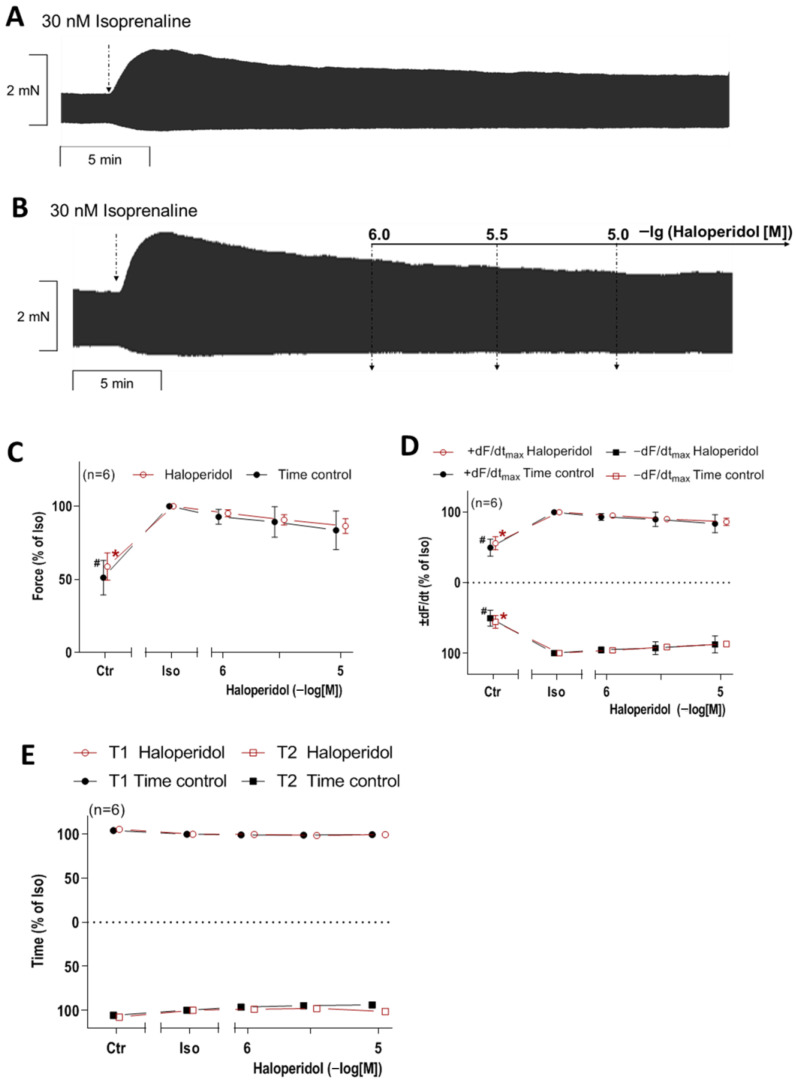
Effects of haloperidol in left atrial preparations of WT in presence of 30 nM isoprenaline. (**A**,**B**) Original recording of inotropic effect of 1, 3, and 10 µM haloperidol on WT mouse left atria in presence of 30 nM isoprenaline (Iso) (**B**) in comparison to time control (**A**). (**C**) Force of contraction: force (100%) = 5.81 ± 1.37 mN; force time control (100%) = 5.22 ± 1.40 mN. (**D**) Maximum rate of tension development (+dF/dt_max_) and maximum rate of relaxation (−dF/dt_max_) in mN/s: +dF/dt_max_ (100%) = 339.99 ± 97.80; +dF/dt_max_ time control (100%) = 294.98 ± 81.01; −dF/dt_max_ (100%) = −213.67 ± 47.33; −dF/dt_max_ time control (100%) = −198.41 ± 54.15. (**E**) Time to peak tension (T1) and relaxation (T2) in ms: T1 (100%) = 18.04 ± 3.51; T1 time control (100%) = 18.22 ± 3.47; T2 (100%) = 29.33 ± 3.58; T2 time control (100%) = 27.09 ± 1.83. * *p* < 0.05 vs. Iso. ^#^ *p* < 0.05 vs. Iso (time control). Control value (Ctr) = pre-drug value. Reading before addition of haloperidol but in presence of isoprenaline was set to 100%. Numbers in brackets indicate number of experiments. Abscissae indicate concentrations of haloperidol in negative logarithmic molar concentrations.

**Figure A3:**
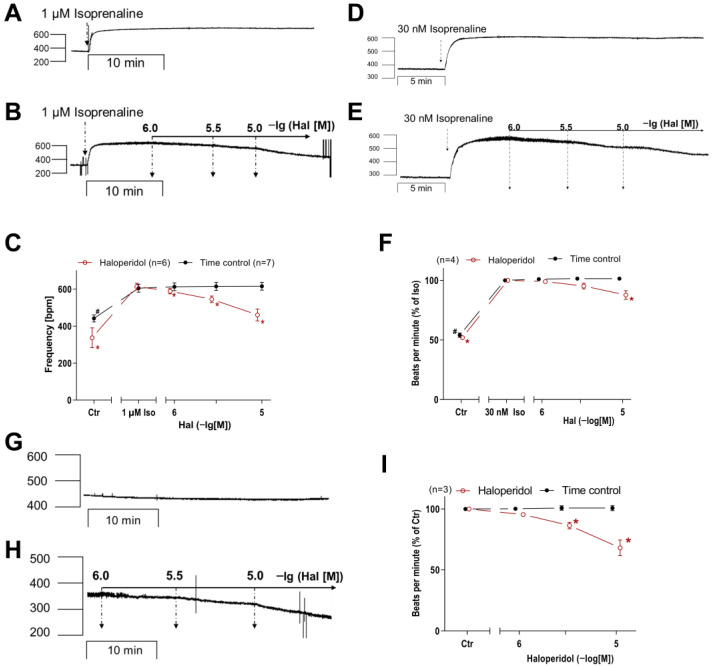
Effect of haloperidol in combination with isoprenaline in right atrial preparations of WT. (**A**,**B**) Original recording of concentration-dependent negative chronotropic effect of haloperidol on WT mouse right atria in presence of 1 µM isoprenaline (**B**) in comparison to time control (**A**). (**C**) Beating rate in beats per minute (bpm): WT (1 µM isoprenaline) = 614.14 ± 18.24; WT time control (1 µM isoprenaline) = 605.12 ± 21.73. (**D**,**E**) Original recording of concentration-dependent negative chronotropic effect of haloperidol in presence of 30 nM isoprenaline (**E**) in comparison to time control (**D**). (**F**) Effects of 1, 3, and 10 µM haloperidol on beating rate in presence of 30 nM isoprenaline (open circle) versus time control (closed circle). Beating rate in beats per minute (bpm): WT (100%) = 610.10 ± 15.40; WT time control (100%) = 585.76 ± 31.19. (**G**,**H**) Original recording of time- and concentration-dependent negative chronotropic effect of haloperidol on WT mouse right atria in Tyrode’s solution (**H**) in comparison to time control (**G**). (**I**) Summary of negative chronotropic effect of haloperidol (open circles) in comparison to time control (closed circles). Where indicated, beating rate before addition of isoprenaline was designated as control value (Ctr). Where indicated, the beating rate before addition of haloperidol was set to 100%. * *p* < 0.05 vs. Iso. ^#^ *p* < 0.05 vs. Iso (time control). Numbers in brackets indicate number of experiments. Abscissae indicate concentrations of haloperidol in negative logarithmic molar concentrations.

In contrast to histamine, which was ineffective in the WT mouse atria (Figure 3), 1 µM isoprenaline increased the FOC in the LA from the WT. Additionally applied haloperidol failed to reduce the FOC (Figure A1B) in comparison to the time control (Figure A1A). The data for the FOC are summarized in Figure A1C. Consequently, haloperidol failed to reduce the maximum rate of tension development and relaxation (Figure A1D), and haloperidol failed to alter the time to peak tension and relaxation in the WT (Figure A1E). The question then arose of whether the concentration of isoprenaline was too high and whether any effects of haloperidol had been overlooked as a result. To this end, a sub-maximal concentration of isoprenaline (30 nM) was used instead in the LA from the WT mice (Figure A2). However, similarly to the results for 1 µM isoprenaline, the haloperidol failed to reduce the FOC stimulated by 30 nM isoprenaline (Figure A2B) in comparison to the time control (Figure A2A). The summarized data for the FOC, rate of tension development and relaxation, and the time to peak tension and relaxation are shown in Figure A2C–E, respectively. Interestingly, there was a regional difference between the effect of haloperidol in LA of WT (Figure A1 and Figure A2) and RA of WT (Figure A3): isoprenaline increased the beating rate in the RA by acting on the sinoatrial node, which was present in the right atrial preparations of the mice as demonstrated in the original recordings (Figure A3A,B,D,E) and in the graphs for 1 µM isoprenaline (Figure A3C) and for 30 nM isoprenaline (Figure A3F). This positive chronotropic effect of isoprenaline could be attenuated by haloperidol after stimulation with 30 nM as well as 1 µM isoprenaline (Figure A3B,C,E,F). In order to complete the study, the effect of haloperidol alone, i.e., in the absence of any pre-stimulation, was investigated (Figure A3G–I). In the original recording (Figure A3H), a negative chronotropic effect in comparison to the time control (Figure A3G) was noted. However, based on the available data, it remains unclear whether this also applies to the cells of the human sinus node (see Section 4).

### 3.5. Influence of the LTCC on the Interaction of Haloperidol and Isoprenaline in the Human Atrial Preparations

Finally, it was investigated whether the LTCC is part of the anti-adrenergic effect of haloperidol in the HAPs, as demonstrated in Figure 4. To this end, 50 mM potassium chloride (KCl) was used. Through this alteration of the transmembrane potential, any increases in the FOC were likely LTCC-mediated. The original recording in Figure 4C demonstrates that the FOC was reduced by the KCl and elevated again by the subsequent addition of 1 µM isoprenaline. The addition of haloperidol reduced the FOC significantly, which is summarized in Figure 4E. This suggests that the effect of haloperidol depends at least in part on its interaction with calcium channels. In Supplementary Figure S6, the effects on the maximum rate of peak tension and relaxation are shown, followed by the time to peak tension and the time of relaxation. Isoprenaline increased the maximum rate of tension development and relaxation, and these parameters were reduced by additionally applied haloperidol (Supplementary Figure S6A). The time parameters were not affected by haloperidol under these conditions (Supplementary Figure S6B).

## 4. Discussion

The primary novel finding of this study is that haloperidol is able to reduce the histamine-induced positive inotropic effect of histamine in the HAPs via H_2_Rs. This finding indicates that haloperidol does not only bind to human H_2_Rs, but acts as a pharmacological antagonist (and not an agonist) of human H_2_Rs in the HAPs. In support of this finding, haloperidol antagonized the positive inotropic and positive chronotropic effects of histamine in the atrial preparations of H_2_-TG. These findings in the mice demonstrate that haloperidol was active as an antagonist of human cardiac H_2_Rs. In this way, the study provides additional functional information about the known biochemical interaction between haloperidol and histamine receptors [11]. In this context, however, it must be taken into account that the expression of the H_2_R in the transgenic mouse model differs from its expression in the human heart [25]. Thus, the employment of the animal model in this investigation is intended less to ascertain the magnitude of haloperidol’s effect on the heart, but rather to explore the possibility of its H_2_R-mediated mechanism.

The utilization of H_2_-TG was useful as a model system because these mice recapitulate many aspects of the human H_2_Rs in the HAPs. The LA from the H_2_-TG were studied in order to facilitate a comparison with the human atrial preparations. In addition, the experiments with the RA from the H_2_-TG also made it possible to study the effect of histamine on the spontaneous beating rate, as has been utilized in previous studies [25]. Transgenic mice (H_2_-TG) have an advantage compared to HAPs in that the experimental conditions can be controlled much better: co-morbidities and medication are not confounders in mouse studies in contrast to HAPs. Nevertheless, the efforts to study HAPs are driven by the wish to offer a translation of the in vitro findings to clinical conditions.

Interestingly, haloperidol on its own reduced the FOC in the HAPs. The exact mechanism for this remains speculative. However, a plausible explanation might lie in the fact that haloperidol can inhibit Ca^2+^ currents through LTCC [12,13], like compounds such as verapamil, which are well known to exert a negative inotropic effect. This observation in the HAPs suggests that haloperidol should be used with caution in patients with heart failure, as there is a concern that it may lead to decompensated heart failure. Furthermore, haloperidol also attenuated the PIE of isoprenaline in the HAPs. This implies an anti-β-adrenergic effect of haloperidol that could be explained by its antagonistic binding to β-adrenoceptors [32]. Here, an anti-β-adrenergic was noted in the mouse sinus node that manifested itself by a reduction in the beating rate. The anti-H_2_-histaminergic effects of haloperidol were less effective than those of cimetidine, a drug that had been tested in H_2_-TG in previous studies [25].

**Figure 5:**
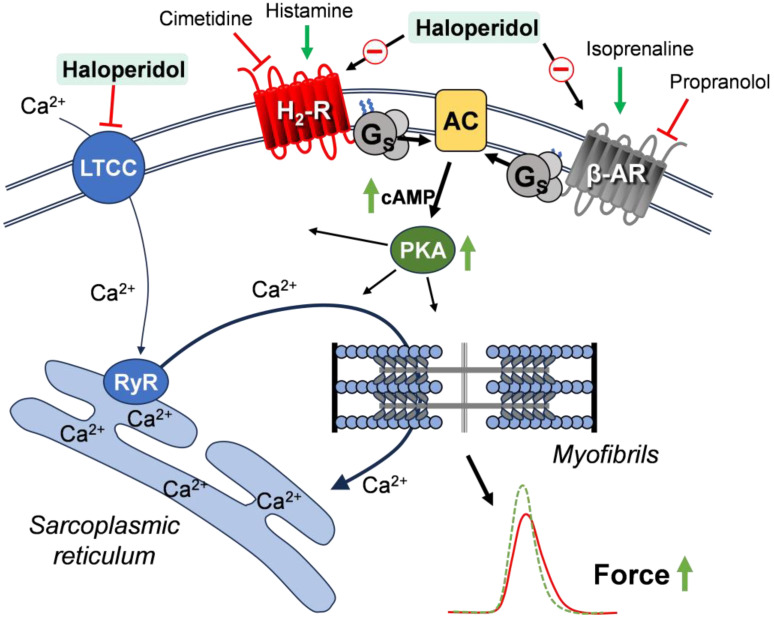
Suggested mechanisms of interaction of haloperidol with histamine H_2_ receptors (H_2_-Rs) and β-adrenoceptors (β-ARs) in cardiomyocytes. Histamine- or isoprenaline-mediated increases in cAMP, PKA activity, and finally force of contraction are indicated by green arrows. AC, adenylyl cyclase; Gs, stimulatory G-protein; LTCC, L-type calcium ion channel; PKA, cAMP-dependent protein kinase; RyR, ryanodine receptor.

The mechanism of the reported cardiac effects is suggested in Figure 5. Haloperidol, by binding to H_2_Rs, reduces the ability of endogenous or exogenous histamine to raise cAMP levels. As a result, the PKA is less activated and less phosphorylation of, e.g., phospholamban, is expected. In this way, haloperidol prolongs the time of relaxation that is shortened by histamine, and reduces the histamine-stimulated FOC. Similarly, the antagonistic action of haloperidol on β-adrenoceptors would reduce the cAMP levels and thus would reduce the efficacy of endogenous noradrenaline to raise the FOC and beating rate. Finally, haloperidol might inhibit, at least in part, the function of the LTCC [12,13]. Thence, less calcium ions could enter the cardiomyocytes and bind to the myofilaments in order to increase the FOC (Figure 5). Additionally, the potential for haloperidol to indirectly influence the activity of ion channels through binding to sigma-1 receptors [33,34] remains a possibility that was not investigated in this study. Of note, the PIE of histamine was only present in the LA from the H_2_-TG, but not in the WT, which is consistent with the initial publication on this mouse model. This is probably due to the very low level of endogenous H_2_R expression in the WT [25]. Likewise, in the RA from the H_2_-TG, a large positive chronotropic effect of histamine was noted. This effect might also occur in humans, because a positive chronotropic effect of histamine has been reported in humans [18], which might be reversed by haloperidol.

The data (Figure 4) for the partially depolarized HAPs provide tentative evidence that haloperidol reduces the FOC under these conditions by attenuating the LTCC function. This could be studied further by performing electrophysiological studies in isolated human atrial cardiomyocytes; however, that was beyond the scope of the present study. In RA of WT mice, haloperidol reduced the beating rate both in the presence (Figure 3) and absence (Figure A3) of propranolol. Therefore, it seems unlikely that β-adrenoceptors were involved in the negative chronotropic effect. Furthermore, histamine was inactive in the WT, so it also seems unlikely that histamine receptors were involved. The effect of haloperidol on sigma-1 receptors could possibly play a role, as sigma-1 receptors are thought to modulate almost all ion channels in the cardiovascular system [33,34]. However, investigating this topic would be a separate project for the future.

There might be detrimental effects of haloperidol by the mechanisms studied. In the context of heart failure, histamine might be an important stimulant of the FOC [18]. Indeed, sufficient production of histamine in the human heart has been observed to enhance contractility in isolated human atrial muscle strips [18]. This stimulatory effect might be counteracted by haloperidol. Haloperidol is metabolized in patients by cytochrome enzymes in the liver [35]. If this metabolism is impeded (genetically or by other drugs), higher plasma concentrations of haloperidol are to be expected, which could increase its anti-histaminergic effects. On the one hand, detrimental effects of haloperidol might occur if it acts similarly as verapamil to inhibit the LTCC, which can induce heart failure by reducing the FOC [36]. On the other hand, the anti-β-adrenergic effects of haloperidol may aggravate heart failure, as has been documented for propranolol, for example [37]. Endogenous catecholamines, such as noradrenaline, can stimulate cardiac β-adrenoceptors and thus raise the FOC in isolated human atriums [23]. These cardio-stimulatory effects might be reduced by haloperidol. Moreover, haloperidol might also alter cardiac function via central mechanisms using hormones. For instance, haloperidol could alter blood pressure via thyroxine and thyrotropin-releasing hormone [38,39]. Other well-known endocrine effects of haloperidol include lactation, even in males, due to a blockade of central D_2_-dopamine receptors and an increased release of hormones (prolactin; [40]).

It is noteworthy that the positive inotropic effect of histamine was finally strongly inhibited by cimetidine after the end of haloperidol administration. Our interpretation is that cimetidine is a more effective antagonist of histamine H_2_ receptors than haloperidol.

### Clinical Relevance and Limitations

The present study demonstrates that haloperidol can principally exert a direct negative inotropic effect. Moreover, haloperidol blocks the positive inotropic effects of three receptors/channels in the human atrium (H_2_Rs, β-adrenoceptors, and LTCC). If haloperidol also blocks these receptors/channels in ventricular tissue, then the clinical impact of this work could be quite relevant. Then, it could be hypothesized that haloperidol, through these mechanisms, may potentially induce or worsen heart failure, particularly when administered in high dosages, for instance, intravenously. In this context, further clinical studies are necessary to investigate the effects in patients with cardiac comorbidities, particularly those treated with high doses of haloperidol. This was, at least, shown in mice, when a transverse aortic constriction was used to decrease cardiac function. Then, chronic treatment with haloperidol led to an enhanced incidence of heart failure and was explained by its toxic effect on mitochondria [41]. At this point, the important limitations of this study must not be concealed: the administration of very high doses (up to 10 µM) of haloperidol in this study, which are never or seldom achieved in practice (the standard therapeutic concentration is typically more than 100-fold lower), could induce unspecific effects in the heart independent of receptor binding. Perhaps this could happen in the event of an overdose, either intentional or unintentional, or as a result of an interaction with other medications, such as liver enzyme inhibitors. On the other hand, the cardiac effects of haloperidol, as measured in vitro, could be compensated for in vivo, for example, by neural counter-reactions. Additionally, the β-adrenergic effects of haloperidol in the heart could be mediated indirectly, rather than by direct binding to β-adrenoceptors. In the past, haloperidol treatment has not been shown to affect the β-adrenergic response, at least in the brain [42]. The influence of the LTCC was only investigated here indirectly by means of cell depolarization using KCl. Electrophysiological investigations would have been a more suitable approach in this case; however, this was not feasible within the scope of the present study.

Another point, likely independent of the receptors studied here, is that haloperidol prolongs the QT interval in a surface ECG; this can lead to ventricular fibrillation, which can be regarded as acute heart failure [43]. Hence, there is clinical evidence that haloperidol can lead to acute heart failure and there is evidence from animal studies that haloperidol can aggravate chronic heart failure, and thence one may speculate that haloperidol might elicit chronic heart failure in patients.

The data observed here from the mouse sinoatrial node do not allow for the conclusion that the same anti-β-adrenergic effect of haloperidol is also present in the human sinoatrial node. Usually, in the mouse heart, less receptors are operational than in the human heart (e.g., here: H_2_Rs). However, if a receptor is found to be active in the mouse heart (e.g., the β-adrenoceptor), it is likely that this receptor is also present in the human sinoatrial node and exhibits a pharmacology analogous to that of the mouse receptor. Research into the effects of haloperidol on the human ventricular myocardium and on the sinoatrial node would be of great interest to investigate possible regional differences in the effects of haloperidol in the human heart, but unfortunately, we do not have access to human sinoatrial node cells or human ventricular preparations. Indeed, histamine has a PIE via H_2_Rs in the human left ventricle [18,20,31]. A further limitation is that we have not constructed complete concentration–response curves for haloperidol and its interaction with isoprenaline. Consequently, a direct comparison of the potency of haloperidol on β-adrenoceptors and histamine H_2_ receptors is not feasible to determine which antagonism is clinically more relevant.

Another limitation of the study is derived from the patient samples. The presence of comorbidities in the patients might have influenced their cardiac responses. However, due to the methodological design of the study, it was not possible to control for these factors. Consequently, on the day of the experiment, it was not known which patients would undergo bypass surgery and had provided informed consent. Therefore, the patients’ clinical data were only available after the experiments had been performed. Furthermore, the experimental data were not adjusted for possible differences between the patients. However, due to the limited number of patients and the high variability between patients, this was not feasible. The fact that several preparations could be obtained from each patient sample was exploited, allowing for the control and effect to be examined on the same patient samples.

In certain instances, the number of cases was very small. This was partly due to the fact that there were not enough human specimens available during the study period, and arrhythmias occurred more frequently in the right atria of mice due to the preparation process, meaning that the specimens had to be discarded. However, when the statistical analysis yielded definitive results, the low number of cases were accepted to avoid the use of additional animals, for ethical reasons, for example.

## 5. Conclusions

In conclusion, haloperidol is an antagonist of human cardiac histamine H_2_ receptors in transgenic mice and in an isolated human atrium. Of course, it should be noted that the cardiac effects of H_2_Rs or other receptors in mice and humans cannot be readily considered equivalent due to species differences. Furthermore, the exact extent of H_2_R expression in mice and patients is unknown and therefore not readily comparable. However, the effects of haloperidol are only relevant at very high doses of haloperidol (1–10 µM, in contrast to the common therapeutic concentration of 40 nM), which could possibly occur in the case of intentional or unintentional overdose or as a result of an interaction with other medications. Thus, interaction of haloperidol, which could lead to negative inotropic and chronotropic effects in the heart, with other QT-prolonging drugs in patients with cardiac comorbidities could potentially lead to cardiac side effects even within the therapeutic range. It is a reasonable assumption that this is a realistic scenario, as haloperidol is a widely used medication, particularly in vulnerable populations. Furthermore, the potential for cumulative effects arises from the interaction of haloperidol with histamine receptors, β-adrenoceptors, and the LTCC. Further clinical studies are needed to confirm the clinical relevance of the observed effects, including an investigation of the effects of haloperidol in patients with comorbidities, where interactions with other medications may occur. An important consequence of this study is that clinical monitoring, including an ECG, should be performed in patients receiving haloperidol, particularly when administered in high doses or when administered intravenously.

## Data Availability

The original contributions presented in this study are included in the article/Supplementary Material. Further raw data supporting the conclusions of this article will be made available by the authors on request.

## Supplementary Materials

The following supporting information can be downloaded at: https://www.mdpi.com/article/10.3390/neurosci6030091/s1, Figure S1: Effect of time and DMSO on force of contraction in right HAPs; Figure S2: Force of contraction in human atrial preparations; Figure S3: Effect of haloperidol in HAPs in presence of histamine; Figure S4: Effects of haloperidol in left atrial preparations of H_2_-TG in the presence of histamine; Figure S5: Effects of haloperidol in HAPs in presence of isoprenaline; Figure S6: Effect of haloperidol in combination with potassium chloride and isoprenaline in right HAPs.

## Abbreviations

The following abbreviations are used in this manuscript:

FOC	Force of contraction
HAP	Human atrial preparation
LA	Left atrial preparation
PIE	Positive inotropic effect
RA	Right atrial preparation
WT	Wild-type mouse

## Appendix A

Further data on the effects of isoprenaline and haloperidol in atrial preparations from wild-type mice.
