# Supplementary material for: Effects of Haloperidol on Cardiac Histamine H2 Receptors and β-Adrenoceptors in Isolated Mouse and Human Atrial Preparations
Source: NeuroSci. 2025 Sep 17;6(3):91. doi: 10.3390/neurosci6030091 (PMC12452768; doi:10.3390/neurosci6030091)
Supplement: Supplementary file 1 [file neurosci-06-00091-s001.zip › neurosci-3805487-supplementary.pdf]

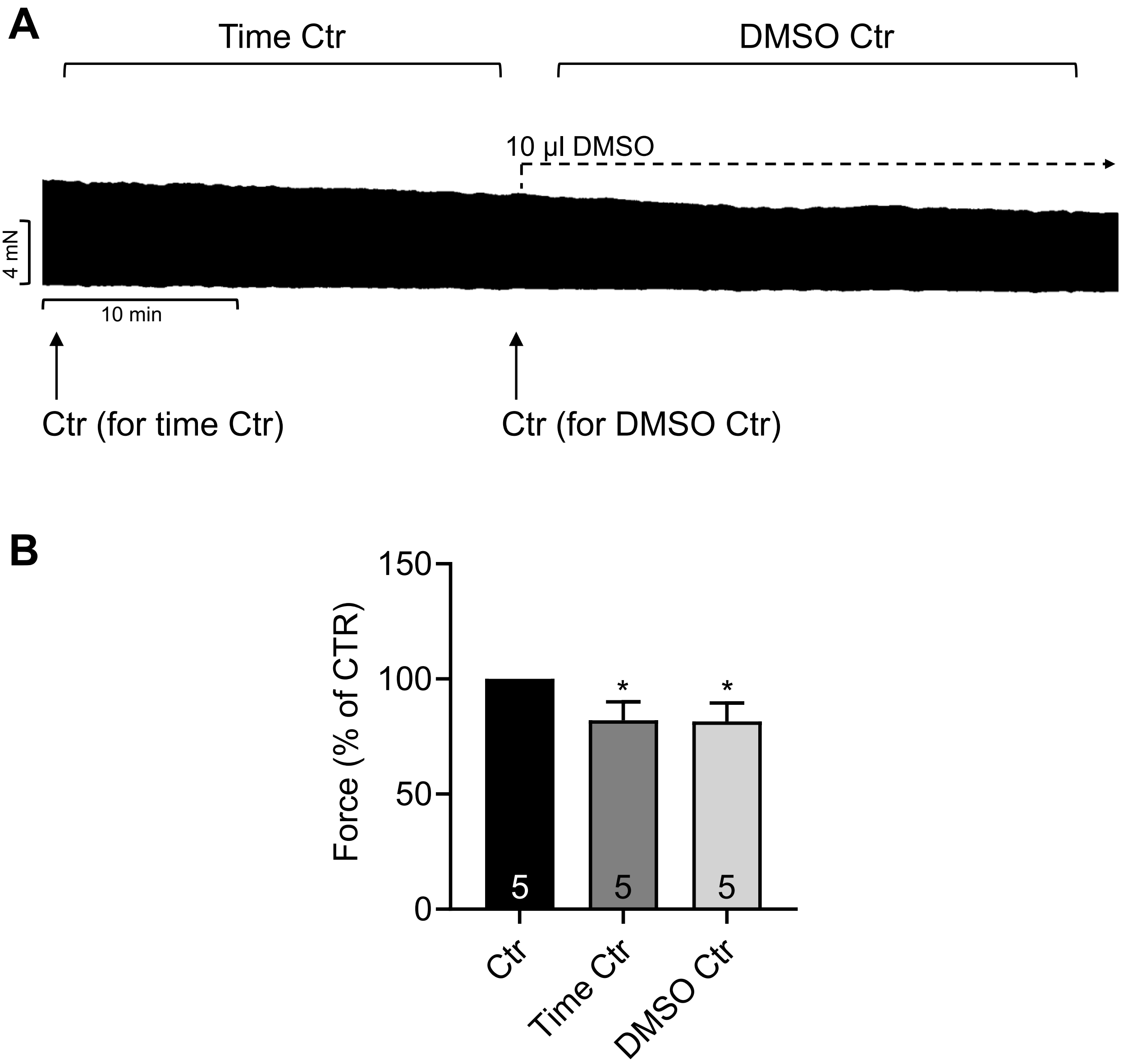

Supplementary Figure S1. Effect of time and DMSO on force of contraction in right HAP. (A) Original recording of a time control (Time Ctr) and a solvent control (DMSO Ctr). **(B)** Summarized data from five control experiments. The force developed before the start of the time control (arrow: for time Ctr) and before the addition of DMSO at the same concentration as for the maximum concentration of haloperidol (arrow: for DMSO Ctr) was set to 100% for the respective subsequent effect. A time frame of 20 minutes was utilized to calculate the time-dependent rundown of the developed force. The numbers in the columns indicate the number of experiments. *$p<0.05$ vs. Ctr.

Figure S2

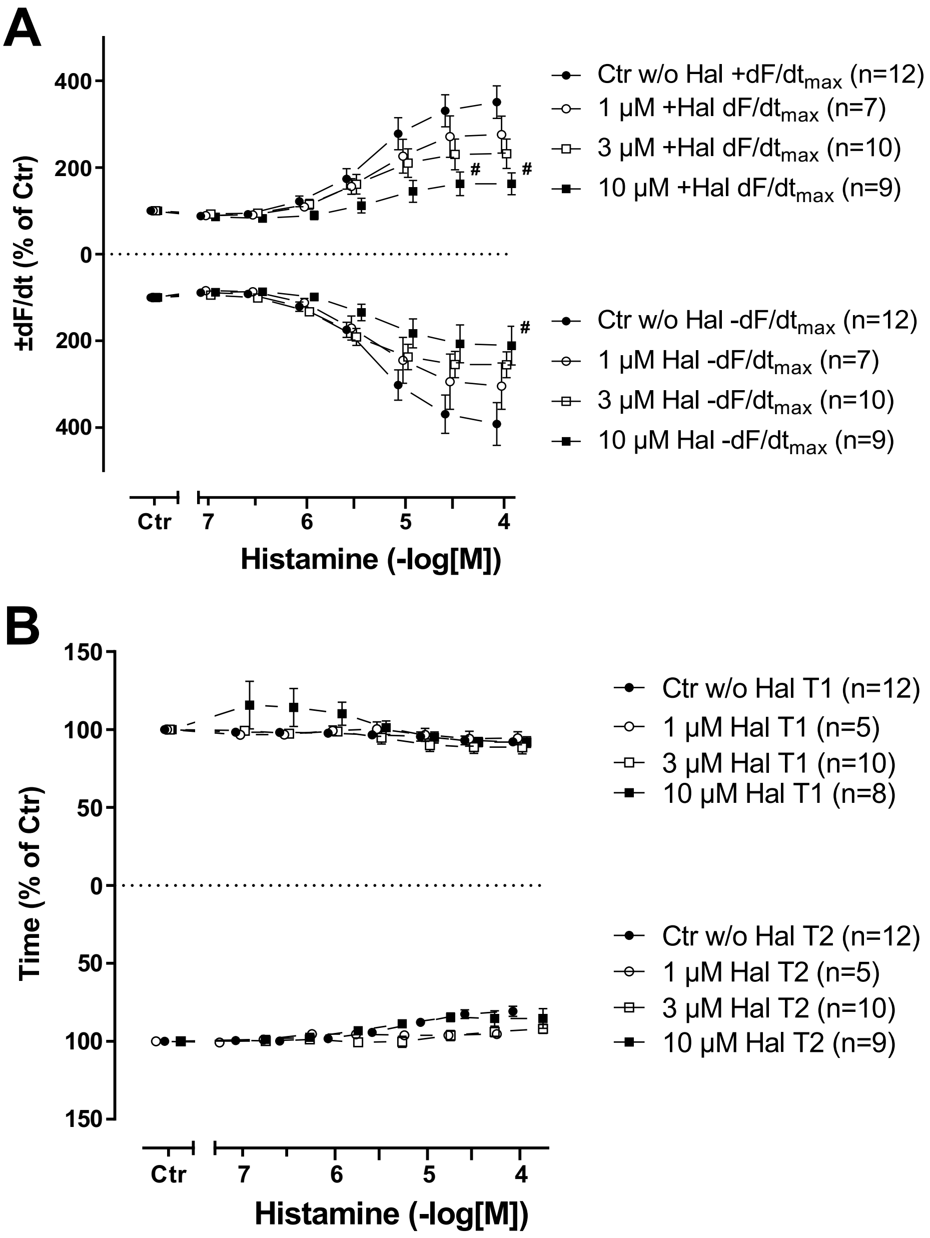

Supplementary Figure S2. Force of contraction in human atrial preparations. The force developed before the addition of histamine, but in the presence of haloperidol where indicated, was designated as the control value (Ctr) and set to 100%. Numbers in brackets indicate number of experiments. *p<0.05 vs. Ctr (pre-histamine values). Abscissae indicate concentrations of histamine in negative logarithmic molar concentrations. **(A)** Maximum rate of contraction (top) and relaxation (bottom)**.** $+dF/dt_{max}$ (100%) = 112.99 ± 21.06 mN/s. $-dF/dt_{max}$ (100%) = -50.36 ± 7.42 mN/s. **(B)** Time to peak tension (top) and relaxation (bottom). T1 (100%) = 54.24 ± 3.41 ms. T2 (100%) = 144.53 ± 7.17 ms.

Figure S3

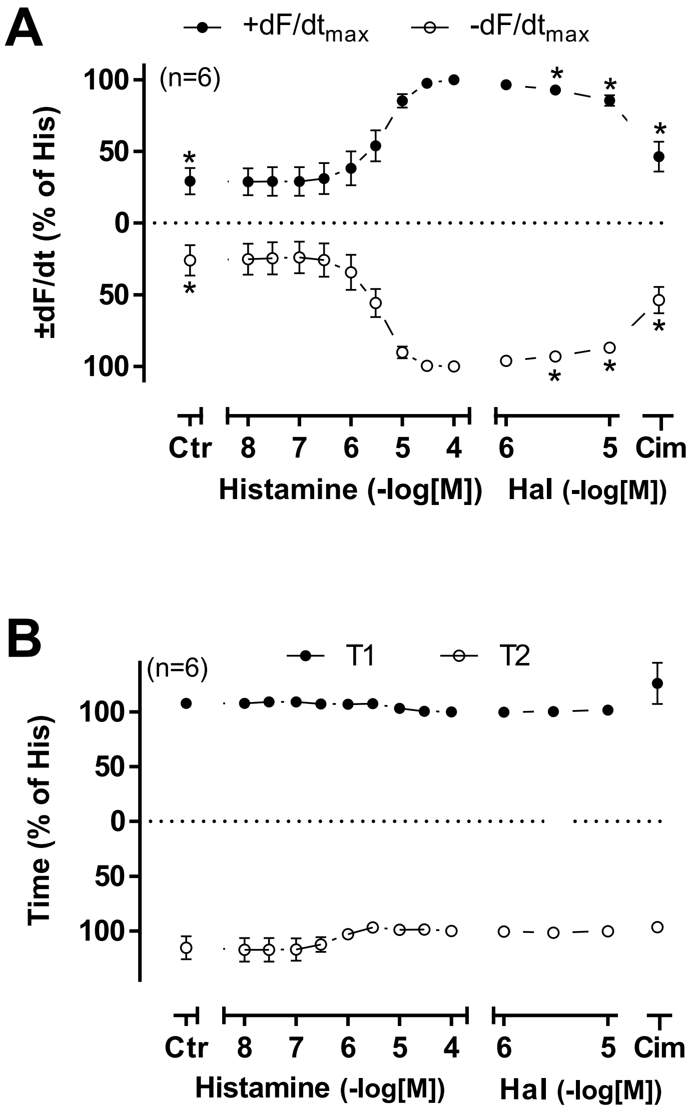

Supplementary Figure S3. Effect of haloperidol in HAP in presence of histamine. Effect of 1, 3, 10 µM haloperidol (Hal) followed by 10 µM cimitidine (Cim) in the presence of 100 µM histamine in HAP. Control values (Ctr) = pre-drug values. The effect before addition of haloperidol but in the presence of histamine was set to 100%. *p<0.05 vs. 100 µM histamine. Numbers in brackets indicate number of experiments. Abscissae indicate concentrations of histamine and haloperidol in negative logarithmic molar concentrations. **(A)** Maximum rate of contraction and relaxation. $+dF/dt_{max}$ (100%) = 203.95 ± 56.87 mN/s. $-dF/dt_{max}$ (100%) = -91.43 ± 16.75 mN/s. **(B)** Time to peak tension and relaxation. T1 (100%) = 48.54 ± 3.96 ms. T2 (100%) = 115.53 ± 6.11 ms .

Figure S4

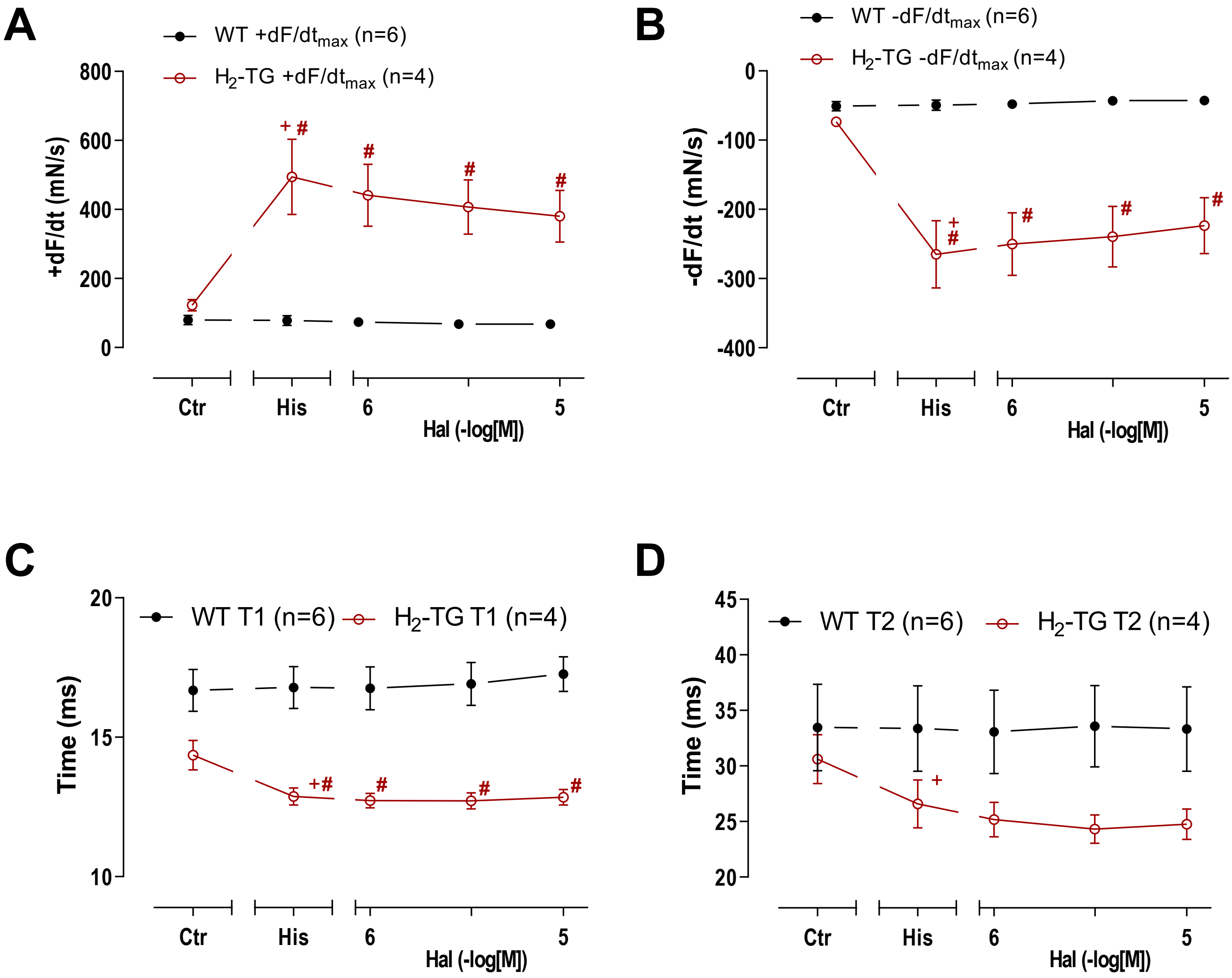

Supplementary Figure S4. Effects of haloperidol in left atrial preparations of H_2-TG in the presence of histamine. Effect of 1, 3, 10 µM haloperidol (Hal) on H_2-TG mouse left atrial preparations in presence of 100 nM histamine (His) compared to wild type (WT) control preparations. The force of contraction before addition of histamine, but in the presence of 0.4 µM propranolol was designated the control value (Ctr). Numbers in brackets indicate number of experiments. Abscissae indicate concentrations of haloperidol in negative logarithmic molar concentrations. **(A)** Rate of tension development (+dF/dt_{max}) and **(B)** rate of relaxation (-dF/dt_{max}). **(C)** Time to peak tension (T1) and **(D)** time to relaxation (T2). Comparison between WT mice (closed circle) and H_2-TG mice (open circle). +$p<0.05$ vs Ctr. *$p<0.05$ vs His 0.1 µM. #$p<0.05$ vs. WT.

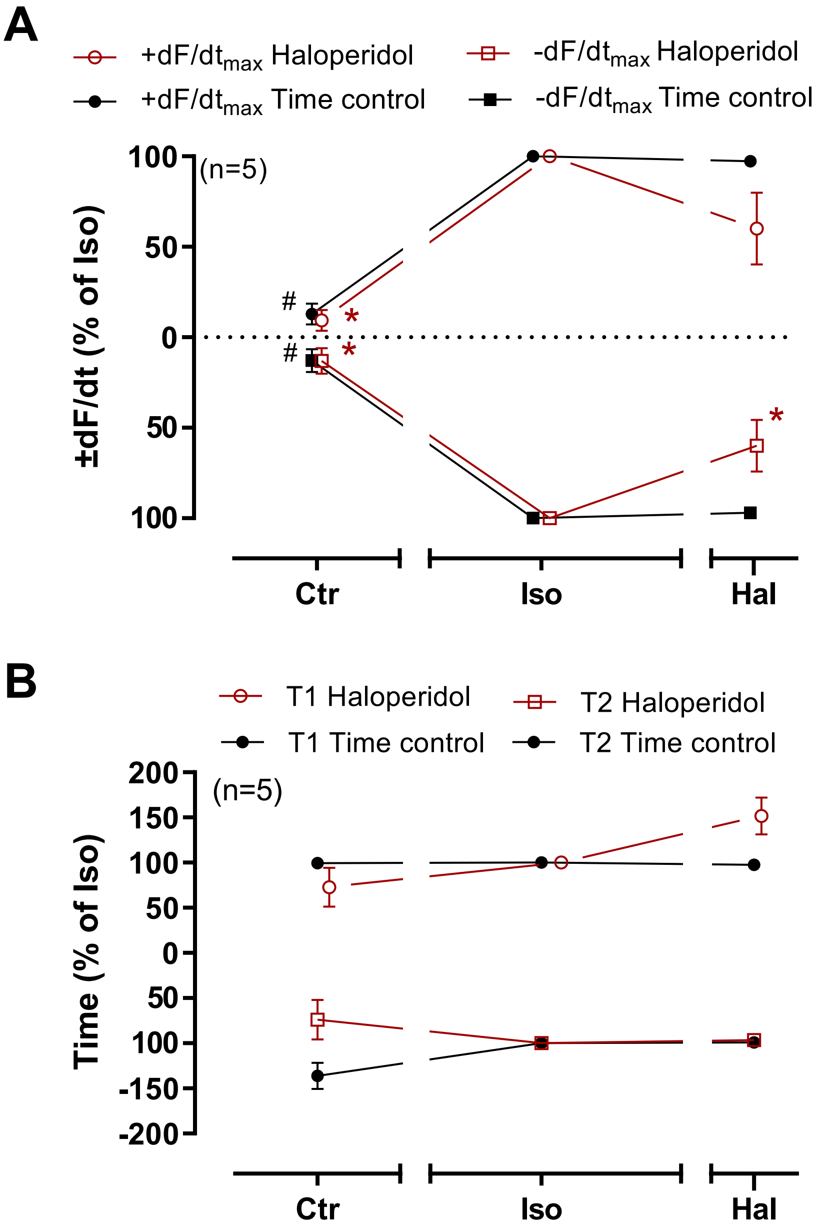

Supplementary Figure S5. Effects of haloperidol in HAP in presence of isoprenaline. Effect of 10 µM haloperidol (Hal) in the presence of 1 µM isoprenaline (Iso) in HAP. Control value (Ctr) = pre-drug value. The effect before addition of haloperidol but in the presence of isoprenaline was set to 100%. *p<0.05 vs. 1 µM isoprenaline. #p<0.05 vs. 1 µM isoprenaline (time control). Numbers in brackets indicate number of experiments. **(A)** Maximum rate of contraction and relaxation. $+dF/dt_{max}$ (100%) = 139.35 ± 50.53 mN/s. $+dF/dt_{max}$ time control (100%) = $-dF/dt_{max}$ (100%) = -72.77 ± 21.10 mN/s. **(B)** Time to peak tension (T1) and relaxation (T2).

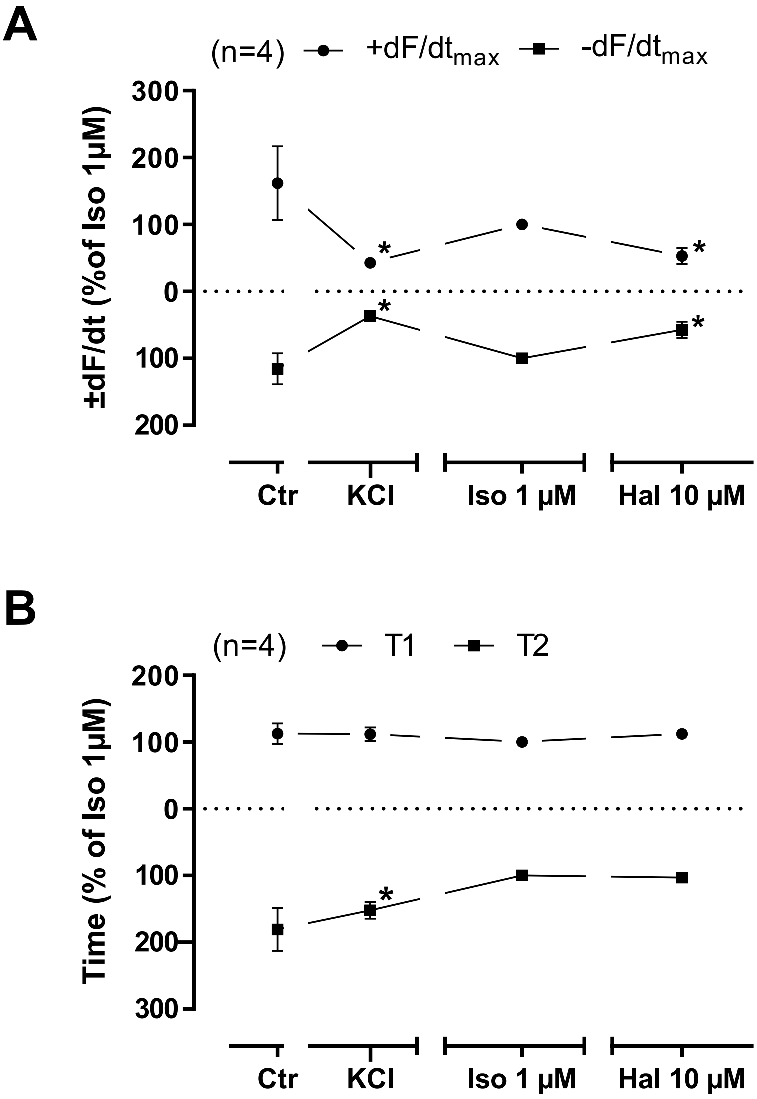

Supplementary Figure S6. Effect of haloperidol in combination with potassium chloride and isoprenaline in right HAP. (A) Maximum rate of contraction (+dF/dt$_{max}$) and relaxation (-dF/dt$_{max}$). +dF/dt$_{max}$ = 77.10 ± 18.84 mN/s. -dF/dt$_{max}$ = -54.74 ± 13.53 mN/s. **(B)** Time to peak tension (T1) and relaxation (T2). T1 (100%) = 49.80 ± 4.03 ms. T2 (100%) = 71.86 ± 3.30 ms. *p<0.05 vs. 1 µM isoprenaline (Iso). Control value (Ctr) = pre-drug value. The developed force before addition of haloperidol (Hal) but in the presence of isoprenaline was set to 100%. Numbers in brackets indicate number of experiments. Abscissae indicate concentrations of haloperidol in negative logarithmic molar concentrations.
